# Excimer 308-nm laser for the treatment of frontal fibrosing alopecia: an observational study in 30 patients

**DOI:** 10.3389/fmed.2026.1736980

**Published:** 2026-06-15

**Authors:** Blanca Ferrer Guillén, Ana Rodríguez-Villa Lario, José María Ricart Vaya, Alba Gómez Zubiaur

**Affiliations:** 1 Trichology Unit, Instituto Médico Ricart, Valencia, Spain; 2 Trichology Unit, Instituto Médico Ricart, Madrid, Spain

**Keywords:** excimer laser 308 nm, frontal fibrosing alopecia (FFA), hair loss (alopecia), laser, narrow band ultra-violet B, scarring alopecia, trichology

## Abstract

**Introduction:**

Frontal fibrosing alopecia (FFA) is a therapeutic challenge. There are few double-blind randomized controlled trials assessing the efficacy of available therapies. The 308-nm excimer laser currently has proven efficacy in treating skin conditions and a potential beneficial effect for the treatment of FFA.

**Method:**

A retrospective observational study was performed to evaluate the efficacy and safety of the 308-nm excimer laser for the treatment of FFA. TrichoScan® was performed at baseline and after 6 months of treatment. Clinical, standardized, and trichoscopic images were taken.

**Results:**

A total of 30 patients (90% women), with a mean age of 65.2 years, were included in the study. All patients had received prior treatments, and the excimer laser was added as an adjuvant treatment in cases with a poor response or a lack of satisfaction; however, no changes were made to their baseline treatment. Clinical response demonstrated stabilization in the recession of the frontotemporal hairline and band-like scarring area in 53.3% of the patients, improvement in 20%, and progression of alopecia in 26.7%. After 6 months of treatment, the majority of patients (83.3%) showed a one-grade improvement in the score of erythema, 6.7% experienced worsened erythema, and erythema reappeared after treatment in 3 patients. The majority of patients (70%) showed a one-grade improvement in the score of perifollicular hyperkeratosis, 10% showed a two-grade improvement, 16.7% remained stable, and hyperkeratosis reappeared after treatment in 1 patient. The only reported adverse effect was transient erythema in 4 patients.

**Conclusion:**

The excimer 308-nm laser might be a promising therapeutic option with a good safety profile for FFA as an adjuvant treatment in patients with symptomatic and progressive disease despite topical and oral therapies.

## Introduction

Frontal fibrosing alopecia (FFA) is a primary lymphocytic scarring alopecia characterized by a progressive recession of the frontotemporal hairline and loss of eyebrows (1). Hair loss is irreversible and causes a major impact on patients’ confidence and quality of life (2). Although its incidence has increased markedly in recent years, the exact origin and pathogenesis remain unclear, with diverse processes that trigger and lead to disease progression. FFA is considered by some authors as a variant of lichen planus pilaris (LPP) with a characteristic clinical pattern (1). However, there are major differences between the two conditions.

The natural history of FFA is variable, and some patients experience a poor response to therapies and disease progression despite being under treatment. FFA is a therapeutic challenge, as there are few double-blind randomized controlled trials assessing the efficacy of available therapies. The current therapeutic strategies are focused on the control of inflammation and fibrosis. The 5-alpha-reductase inhibitor dutasteride and intralesional corticosteroids have been positioned as the most effective treatments to date, followed by topical corticosteroids and calcineurin inhibitors, antimalarials, and, more recently, topical and systemic Janus kinase (JAK) inhibitors (2).

Excimer laser is a subtype of narrowband ultraviolet B light (NB-UVB) that emits a 308-nm wavelength and is widely used throughout the field of dermatology for different conditions. This light is absorbed by keratinocytes, T-cells, and chromophores such as DNA, causing DNA damage while reducing local inflammation and keratinocyte proliferation. It also upregulates the p53 tumor suppressor pathway and downregulates the Bcl-2 proto-oncogene, leading to cell arrest and apoptosis. This mechanism translates into a combined action of inhibition of dermal proliferation and an anti-inflammatory effect through lymphocyte apoptosis. It also exerts immunomodulatory effects, including inhibition of antigen-presenting cells, modification of the Th1 phenotype toward a Th2 one, and induction of regulatory T-lymphocytes (3, 4).

The excimer 308-nm laser has been used successfully in the treatment of skin conditions such as vitiligo, psoriasis, atopic dermatitis, alopecia areata, allergic rhinitis, folliculitis, granuloma annulare, lichen planus, mycosis fungoides, palmoplantar pustulosis, pityriasis alba, CD30 + lymphoproliferative disorder, leukoderma, prurigo nodularis, localized scleroderma, and genital lichen sclerosus (3, 4), and it has a potential beneficial effect for the treatment of frontal fibrosing alopecia.

## Method

A retrospective observational study was performed to evaluate the efficacy and safety of the 308-nm excimer laser for the treatment of FFA. A total of 30 patients with FFA who were treated with the 308-nm excimer laser were included. The demographic and clinical characteristics were assessed. TrichoScan® was performed at baseline and after 6 months of treatment. Clinical standardized macroscopic images were taken, and clinical response was evaluated by two independent dermatologists. Six measurements in centimeters were taken at baseline and after 6 months of treatment. Three measurements evaluated the recession of the frontotemporal hairline from the glabellar midpoint and the outer canthus of the right and left eyes, respectively. Three measurements evaluated the band-like scarring area or hair loss area in the center of the forehead and in the lines above the right and left pupils. Three trichoscopic images were obtained from the middle, right, and left regions of the frontotemporal hairline. The grade of inflammation was evaluated in the trichoscopic images at baseline and after 6 months of treatment, based on the severity of erythema and hyperkeratosis in three grades (no, mild, or severe). Adverse effects were assessed, and their management or need for intervention was determined. A survey was administered to patients to assess the subjective efficacy of treatment on symptoms and activity in FFA and their perceptions of treatment innovation.

## Results

The mean age of patients was 65.2 years (range: 47–80), and 90% were women. The mean time of evolution of the disease was 6 years (range: 2–25). The majority of patients (73.3%) had pattern I or “linear pattern” alopecia, 20% had pattern II or “diffuse pattern” (20%), and 6.7% had pattern III or “pseudo-fringe-sign pattern.” Overall, 24 of the 30 patients had eyebrow loss; 63.3% had greater than 75% to complete loss of eyebrows, 16.7% had partial eyebrow loss (between 25 and 75%), and 20% had no involvement or less than 25% eyebrow loss. All patients had received prior treatment, and the 308-nm excimer laser was added as an adjuvant therapy in cases with a poor response due to pruritus or clinical or trichoscopic signs of activity or lack of satisfaction with previous therapy. No changes were made to their baseline treatment regimen during the preceding 6 months or at the time the excimer laser was added. Baseline treatment consisted of topical corticosteroid (83.3% of patients), mesotherapy with corticosteroid (90%), oral dutasteride (86.7%), and oral minoxidil (6.7%).

The initial dose of the 308-nm excimer laser was 250 millijoules (mJ)/cm^2^ in all patients, with dosage increments of 50 mJ/cm^2^ per session if tolerated, up to a maximum dose of 400 mJ/cm^2^. The mean number of sessions received per patient was 12, with a range from 3 to 14. The majority of patients (43.3%) received the 308-nm excimer laser every 3 weeks, followed by 23.3% of patients receiving treatment every 2 weeks and 23.3% of them every 4 weeks, and 10.1% of them every 6 weeks. The mean dose received per patient was 5,254 mJ/cm^2^ after 6 months of treatment.

Clinical response after 6 months of treatment with the 308-nm excimer laser demonstrated stabilization in recession of the frontotemporal hairline and the band-like scarring area in 53.3% of patients, improvement in 20%, and progression of alopecia in 26.7%.

Trichoscopic parameters at baseline and after 6 months of treatment are shown in Table 1. The majority of patients (83.3%) showed a one-grade improvement in erythema score, 6.7% worsened, and erythema reappeared after treatment in 3 patients. The majority of patients (70%) showed a one-grade improvement in the perifollicular hyperkeratosis score, 10% showed a two-grade improvement, 16.7% remained stable, and hyperkeratosis appeared after treatment in 1 patient.

**Table 1:** Trichoscopic parameters at baseline and after 6 months of treatment.

Trichoscopic parameters	Baseline	After 6 months
Perifollicular erythema		
Absence	10%	63.3%
Mild	56.7%	33.3%
Severe	33.3%	3.4%
Perifollicular hyperkeratosis		
Absence	3.3%	46.6%
Mild	43.4%	33.3%
Severe	53.3%	10%

The reported adverse effect was transient erythema in 4 patients, all of which was mild and resolved without the need for dose reduction or interruption of treatment. The erythema appeared in session numbers 2 (dose 300 mJ/cm^2^), 7 (dose 300 mJ/cm^2^), 4 (dose 400 mJ/cm^2^), and 15 (dose 400 mJ/cm^2^), respectively. In these cases, the dose was not increased; however, patients continued the 308-nm excimer laser therapy, and the erythema resolved without any treatment. There were no cases of hyperpigmentation or other adverse effects.

Subjective efficacy perceived by patients in the symptoms and activity in FFA after 6 months of adjunctive treatment with the 308-nm excimer laser indicated worsening (20%), no change (16.7%), slight improvement (26.7%), moderate improvement (26.7%), and great improvement (9.9%). On a scale of 1 to 5, where 1 corresponds to no innovation and 5 corresponds to great innovation, patients rated the 308-nm excimer laser as 1 (10%), 2 (13.3%), 3 (33.3%), 4 (30%), and 5 (13.4%) in terms of perceived treatment innovation.

The excimer laser was discontinued in 5 patients (16.7%) due to a lack of efficacy.

## Discussion

Several studies have hypothesized that the 308-nm excimer laser might also be effective in LPP and FFA, since it has proven beneficial in certain inflammatory skin disorders mediated by lymphocytes and chronic inflammation.

Navarini et al. (5) include the cases of 13 patients with LPP or its variants, including frontal fibrosing alopecia, who were treated twice weekly up to a total mean dose of 4,300 mJ/cm^2^. After laser therapy, they found a significant reduction in inflammatory activity in their patients, expressed by decreased erythema, pain, pruritus, and hyperkeratosis.

Fertig et al. (6) observed that the most effective treatment for FFA in their large cohort of patients in clinical practice was achieved with a combination of oral finasteride, hydroxychloroquine, topical calcineurin inhibitors (tacrolimus), and excimer laser.

Zhang et al. (7) analyzed characteristics and treatment in 29 patients with FFA, of whom 3 were treated with the 308-nm excimer laser in combination with other therapies, primarily topical calcineurin inhibitors, topical and intralesional corticosteroids, and hydroxychloroquine. One of the 3 patients showed regrowth, and 2 patients remained stable. The abovementioned authors concluded that the excimer laser was among the most efficacious treatments for limiting hair loss and was effective in reducing inflammatory symptoms.

Recent updates in the management strategies and efficacy of treatment modalities, such as those published by Gamret et al. (8) and Starace et al. (9), refer to the 308-nm excimer laser as one of the therapies that can be useful in patients with an active inflammatory component, as it is effective in reducing inflammation and peripilar casts.

To date, scientific evidence for the use of the excimer laser in the treatment of FFA remains limited, as published studies have reported isolated cases of FFA within series of LPP, although the results indicate potential benefits.

To the best of our knowledge, our study represents the largest series of patients with FFA who were treated using the 308-nm excimer laser.

The limitations of our analysis are the small sample size, retrospective design, and potential bias in the evaluation of the efficacy of the excimer laser due to concomitant therapies. All patients received systemic and topical treatments. However, the aim of our study was to evaluate the efficacy and safety profile of the excimer laser in these conditions as an adjuvant treatment in FFA.

In conclusion, the 308-nm excimer laser might be a promising therapeutic option with a good safety profile for FFA as an adjuvant treatment in patients with symptoms and disease progression, despite adequate therapy. It has been shown to reduce inflammation, as reflected by improvement in perifollicular erythema and, especially, hyperkeratosis. No serious adverse effects were reported.

Future research is needed to establish the optimal treatment protocol and the positioning of the excimer laser in the FFA therapeutic algorithm.

## Data Availability

The original contributions presented in the study are included in the article/supplementary material, further inquiries can be directed to the corresponding author.
